# Examining validity of body mass index calculated using height and weight data from the US driver license

**DOI:** 10.1186/s12889-019-6391-3

**Published:** 2019-01-22

**Authors:** Alla Chernenko, Huong Meeks, Ken R. Smith

**Affiliations:** 1 Department of Sociology, University of Utah, 390 South 1530 East, Rm 301, Salt Lake City, UT 84112 USA; 2 Population Sciences, Huntsman Cancer Institute, University of Utah, 2000 Circle of Hope, Salt Lake City, UT 84112 USA; 3 Department of Family and Consumer Studies, University of Utah, 225 South 1400 East Alfred Emery Building 228, Salt Lake City, UT 84112 USA

**Keywords:** Body mass index, Self-report, Bias, Driver license

## Abstract

**Background:**

Driver license departments in many US states collect data on individuals’ height and weight. These data can be useful to researchers in epidemiological and public health studies. As height and weight on driver license are self-reported, they may be prone to reporting bias. We compare height and weight obtained from driver license records and clinically measured height and weight, as well as body mass index (BMI) values calculated using the two data sources for the same individual.

**Methods:**

We linked individual height and weight records obtained from the Driver License Division (DLD) in the Utah Department of Public Safety to clinical records from one of the largest healthcare providers in the state of Utah. We then calculated average differences between height, weight and BMI values separately for women and men in the sample, as well as discrepancies between the two sets of measures by age and BMI category. We examined how well self-reported height and weight from the driver licenses classify individuals into specific BMI categories based on clinical measures. Finally, we used two sets of BMI values to estimate individuals’ relative risk of type II diabetes.

**Results:**

Individuals, on average, tend to overestimate their height and underestimate their weight. Consequently, the value of BMI calculated using driver license records is lower than BMI calculated using clinical measurements. The discrepancy varies by age and by BMI category. Despite the discrepancy, BMI based on self-reported height and weight allows for accurate categorization of individuals at the higher end of the BMI scale, such as the obese. When used as predictors of relative risk of type II diabetes, both sets of BMI values yield similar risk estimates.

**Conclusions:**

Data on height and weight from driver license data can be a useful asset for monitoring population health in states where such information is collected, despite the degree of misreporting associated with self-report.

## Background

Body mass index (BMI) is an important biometric measure commonly used across numerous disciplines to assess risk of many health conditions. Increased BMI is associated with excess health risks, including insulin resistance and hyperinsulinemia, Type II diabetes mellitus, hypertension, dyslipidemia, coronary heart disease, asthma, arthritis, gallbladder disease, several cancers, depression, as well as with increased all-cause mortality [1–8]. Individuals classified as underweight based on their BMI also experience heightened health and mortality risks and are likely to have poor psychological health [4, 5, 9]. While commonly used in clinical practice and public health research, BMI is not necessarily a perfect predictor of individual health. Multiple studies highlight limitations of BMI in certain subpopulations including children, teenagers, elderly and ethnic minority patients, and suggest the use of alternative anthropometric indicators. These include waist circumference, waist-to height ratio, waist-to-hip ratio, percent body fat, and fasting leptin levels, which may be more useful for predicting adiposity and associated health risks than BMI [7, 10–17]. Alternative anthropometric measures have been used to supplement BMI to refine health risk estimates within BMI categories [18].

One of the primary advantages of using BMI in population health research is not only its centrality to key biological pathways leading to crucial health outcomes, but also that it is relatively straightforward to measure. Many studies rely on self-reported height and weight to calculate BMI, and while these measures are prone to reporting biases, they are the best available option in larger-scale studies where direct measurement of height, weight or other anthropometric characteristics is difficult or prohibitively expensive.

Several studies document the existing discrepancies between self-reported height and weight and their clinically measured counterparts, as well as their derived BMI values [19–34]. Research demonstrates that individuals in general tend to overestimate their height and underestimate their weight, although the degree of discrepancy varies across different demographic categories. For example, increasing age is associated with more disagreement between self-reported and measured height and weight, likely due to changes in stature and body composition and illnesses common among older individuals [19, 20]. Misreporting of weight also varies by ethnicity and is more prevalent for individuals at the lower and higher ends of the BMI spectrum [7, 20, 22, 23, 35–37]. In addition, certain behaviors and medical conditions can play a role, as individuals with a history of dieting may be more likely to underestimate their weight, while people with history of eating disorders are more accurate in their reporting compared to the general population [20, 38]. Despite the discrepancies, several studies generally show high correlation and agreement when comparing self-reported and clinically measured values [19, 21, 24, 32, 33, 39, 40]. Given the bias towards lower BMI values, the association between BMI and certain health and mortality risks is likely to be biased, perhaps underestimated, when BMI measure is derived from self-reported height and weight [39, 41]. Some authors suggest correcting for the measurement error when using BMI based on self-reported height and weight when estimating health and mortality risks, noting that although corrected BMI performs better than uncorrected BMI, these corrections do not eliminate the bias completely [42]. BMI derived from self-reported height and weight should be therefore treated with caution, yet it nonetheless remains an essential measure in epidemiological studies.

In the US, height and weight information is collected in many states by their respective driver license or motor vehicle departments. In some instances, these data offer researchers an opportunity to use height and weight data from the driving public for medical and public health investigations. Although access to driver license data in some states may be heavily restricted, it can be possible for public health researchers to obtain millions of individual records from appropriate governmental agencies responsible for maintaining the driver license records in different states [43, 44]. Since height and weight information contained in the driver license records is self-reported, it is likely prone to errors relative to clinical measures similar to those found in surveys that too rely on self-reported anthropometric information. Ossiander, Emanuel, O’Brien and Malone [45] linked 480 records from women enrolled in a population-based cancer etiology study in Washington state to their driver license records. They reported high positive correlations for both height and weight reported on the driver license and when measured during the study, despite the average discrepancies of 0.28 cm for height and 5.8 kg for weight, generally with height being overestimated and weight being underestimated on the driver license. An earlier study of a sample of 140 Asian women in Hawaii found that individuals underestimated their weight by 4.74 kg and overestimated their height by 2.06 cm [46]. Morris et al. [47] used driver license data for the state of Oregon to estimate BMI at the Census block group level and compared the estimates to those obtained from the Oregon Behavioral Risk Factor Surveillance System (BRFSS) – a CDC statewide random-digit-dial telephone survey. Although BRFSS also relies on self-reported height and weight data, block group level obesity prevalence calculated based on the driver license data was 18% lower than BRFFS for men and 31% lower for women. At the same time, average Census block group BMI estimates showed a more modest discrepancy of 2% for men and 5% for women, with values derived from the driver license data being lower than those obtained from BRFFS. The results of these studies suggest that although information about height and weight obtained from driver licenses introduce reporting errors, it is biased in a predictable manner, i.e. height is likely overestimated, weight is likely underestimated, and, consequently, BMI calculated using these height and weight values is underestimated.

In this study, we examined the disagreement between BMI calculated using height and weight measured clinically and captured in electronic medical records and BMI calculated using height and weight obtained from driver licenses in a large sample of Utah drivers. We then evaluated the utility of the driver license and clinically measured height and weight by estimating the relative risks of having Type II diabetes using the two alternative versions of BMI as predictors to assess the usefulness of driver license data on height and weight in public health studies.

## Methods

### Data

The height and weight data were obtained from two sources. First, we used height and weight data provided to the Utah Population Database (UPDB) by agreement from the Driver License Division (DLD) in the Utah Department of Public Safety. Annual updates of driver license information from the DLD are linked to the UPDB. Second, height and weight data were also available from the University of Utah Health Science Center (UUHSC) – one of the two largest healthcare providers in the state of Utah – which maintains all clinical records for patients seen at the UUHSC, including anthropometric measures. These UUHSC records are linked to the UPDB at the individual level and are updated every six months.

From the Utah Population Database (UPDB), we selected 33,354 individuals with height and weight data from Driver License Division and University of Utah Health Science Center. We then restricted the sample to individuals who had complete height and weight values from both DLD and UUHSC, BMI values calculated from both sources between 12 and 60 kg/m^2^, and differences in height and weight values between two sources not exceeding 10 cm and 40 kg respectively. We selected the cut-off for the difference between self-reported and clinically measured height based on the literature [32]. We were not able to establish a weight difference cut-off the same way, and opted for a data-driven approach, eliminating cases where difference between self-reported and clinically measured weight values were four or more standard deviations away from the mean. Using these cut-off points, we were able to allow for variation in values, while omitting the more extreme differences.

We also required that the dates on which clinical height and weight were measured were within 365 days of the dates on which height and weight were reported on the driver license, excluding individuals with larger gaps between the dates the measurements were reported. Finally, we excluded individuals whose last available follow up dates in UPDB were less than one year from when the height and weight were measured by UUHSC. While some individuals in this category have been lost to follow up, others died within 365 days after their UUHSC visit, which means they may have been severely ill at the time of the visit, and the illness, in turn, may have affected their weight. The final study sample comprised 16,576 subjects.

### Analysis

We generated sex-specific descriptive statistics for the sample to illustrate the height and weight characteristics in the DLD (height_D_ and weight_D_) and clinical records (height_C_ and weight_C_), as well as BMI values calculated using the height and weight from the two different sources (BMI_D_ and BMI_C_). BMI categories are defined as follows: underweight (BMI < 18.5 kg/m^2^), normal weight (BMI between 18.5 and 24.9 kg/m^2^) overweight (BMI between 25.0 and 29.9 kg/m^2^), Class I (BMI between 30.0 and 34.9 kg/m^2^) and Class II - Class III obesity combined (BMI ≥ 35 kg/m^2^). Formal Class III obesity individuals were too few in number to be treated as a separate category.

We then calculated differences between the mean DLD and clinical height (height_D_ – height_C_), DLD and clinical weight (weight_D_ – weight_C_), and the BMI values calculated using DLD and clinical sources (BMI_D_ – BMI_C_) overall and by Differences were calculated separately for individuals of different ages (based on age provided in DLD records) and different BMI categories (based on BMI_c_). Negative difference values indicate underestimation in the DLD values compared to the clinical values obtained from the UUHSC, and positive difference values reflect overestimation in the DLD values. Paired Wilcoxon signed rank tests were used to evaluate the differences between mean height, weight and BMI. This allowed us to understand the extent of variation in misreporting of height and weight by age and BMI. In this case, the paired Wilcoxon signed rank tests were selected because, while the two sets of measures being compared were obtained from the same sample of individuals, the distribution of differences between the two sets of measures were not normally distributed, hence warranting a non-parametric test. We also established that the variances in the two sets of measures were unequal, with few exceptions.

Cross-classifications of BMI_D_ and BMI_C_ were performed to determine to what extent self-reported height and weight from the driver licenses allows to classify individuals into specific BMI categories. Finally, we used logistic regression models to estimate individuals’ likelihood of having type II diabetes using BMI_C_ and BMI_D_ as key predictors and controlling for birth year, sex, level of education, race and ethnicity. Four models were estimated using continuous and categorical BMI_C_ and BMI_D_ as predictors. Information on individuals’ diabetes diagnosis were obtained from statewide inpatient discharge and ambulatory surgery records for individuals seen at UUHSC, all of which are linked to UPDB. All analyses were performed using R statistical software version 3.4 (https://www.r-project.org/).

## Results

Descriptive characteristics of the sample are presented in Table 1. Among both men and women, average values of height as reported in the DLD records exceed those found in clinical records, and values of weight_D_ are smaller than values of weight_C_. Average height for women in our sample is 163.8 cm based on the clinical data, and 164.1 cm based on the DLD data. Average weight for women, as reported in the UUHSC data, is equal to 79.1 kg, and their average weight based on the DLD records is 73.4 kg. For men, average values of height_C_ and height_D_ are 178.4 cm and 178.9 cm accordingly, while average weight_C_ for men is 94.4 kg, compared to the average value of 91.6 kg for weight_D_. Consequently, average BMI_D_ calculated using the DLD records is lower than BMI_C_ calculated using clinical records from the UUHSC for individuals in our sample. Among women the BMI discrepancy is equal to − 2.19 kg/m^2^, while among men the difference between BMI_D_ and BMI_C_ is − 1.06 kg/m^2^. Sex-specific height, weight and BMI differences are statistically significant at *p* ≤ 0.001.

**Table 1:** Descriptive characteristics of the sample

	*Female* *(N = 8905)*		*Male* *(N = 7671)*	
	DLD	Clinical	DLD	Clinical
Height (cm)	164.1 ± 7.0	163.8 ± 7.0	178.9 ± 7.7	178.4 ± 7.7
Weight (kg)	73.4 ± 18.5	79.1 ± 21.0	91.6 ± 20.2	94.4 ± 21.8
BMI (kg/m^2^)	27.3 ± 6.7	29.5 ± 7.6	28.6 ± 5.7	29.6 ± 6.2
*BMI categories (%)*				
Underweight	231 (2.6)	191 (2.1)	51 (0.7)	48 (0.6)
Normal weight	3741 (42.0)	2742 (30.8)	2035 (26.5)	1738 (22.7)
Overweight	2387 (26.8)	2306 (25.9)	3032 (39.5)	2762 (36.0)
Type I obesity	1428 (16.0)	1718 (19.3)	1628 (21.2)	1814 (23.6)
Type II/III obesity	1118 (12.6)	1948 (21.9)	925 (12.1)	1309 (17.1)
Age (years)	49.0 ± 17.4	49.3 ± 17.4	51.9 ± 17.4	52.2 ± 17.4
*Race (%)*				
White	7764 (87.2)		6613 (86.2)	
Other	497 (5.6)		415 (5.4)	
*Ethnicity (%)*				
Hispanic	1300 (14.6)		902 (11.8)	
Non-Hispanic	5848 (65.7)		5186 (67.6)	
*Education (%)*				
Less than high school	590 (6.6)		407 (5.3)	
High school	1798 (20.2)		1301 (17.0)	
Some college	1954 (21.9)		1455 (19.0)	
College graduate	961 (10.8)		883 (11.5)	
Graduate/professional degree	714 (8.0)		1120 (14.6)	

*Note.* Mean values and standard deviations are reported for continuous variables: height, weight, BMI and age. For categorical variables – categorical BMI, race and education – number of individuals in each category is reported and corresponding percentage is presented in parentheses

**Fig. 1:**
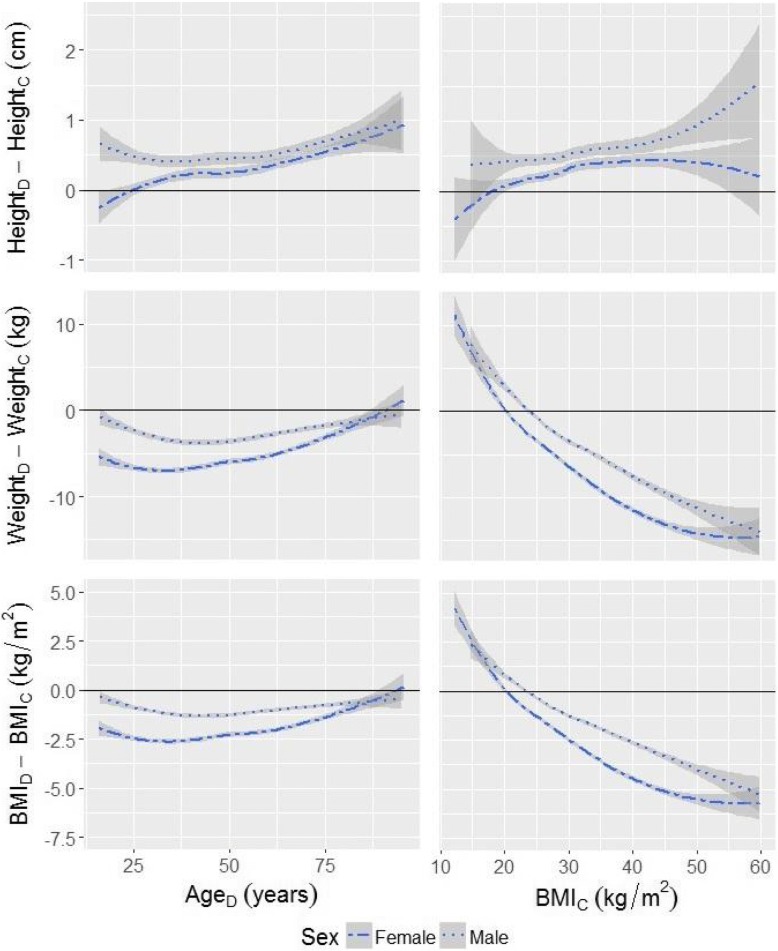
Distribution of discrepancies between height, weight and BMI values obtained from the driver license records and clinically measured values by age and BMI_C_

Height, weight and BMI differences vary among individuals based on age and clinical BMI value (Fig. 1). For women in our sample, the difference between height_D_ and height_C_ appears to increase with age, with older women having, on average the largest difference between the values. Women between the ages of 25 and 34 overestimate their height_D_, on average, by 0.13 cm, while women 65 years old and older report height_D_ that exceeds their clinically measured height_C_ by 0.52 cm. Average differences for other age group fall between these values, with the exception of women between the ages of 16 and 24, who underestimate their height by an average of − 0.6 cm. Women between the ages of 16 and 24 are also the only category, for whom the difference between average clinical and self-reported height values is not statistically significant. Conversely, younger women, on average, underestimate their weight on the driver license to a greater extent than older women, with those between the ages of 25 and 34 reporting weight_D_ values that are, on average, 6.98 kg lower than their clinically recorded weight_C_ values. The difference diminishes with age. A similar pattern is observed with regard to BMI_D_ and BMI_C_, with BMI_D_ − BMI_C_ = − 2.61 kg/m^2^ for women between the ages of 25 and 34, and BMI_D_ − BMI_C_ = − 1.32 kg/m^2^ for women 65 years old and older, with average differences for the remaining age groups falling between the two extremes.

Among men, the largest differences between average height_D_ and height_C_ are observed in ages 16 to 24 (height_D_ − height_C_ = 0.63 cm) and after age 65 (height_D_ − height_C_ = 0.68 cm). At the same time, men in these age groups have the lowest differences between average weight_D_ and weight_C_ (− 1.45 kg for men between the ages of 16 and 24 and − 1.86 kg for men 65 and older). Those between the ages of 35 and 44 have the largest difference between average weight_D_ and weight_C_, underestimating their weight by 3.91 kg. Although the degree of misreporting varies, men tend to overestimate their height and underestimate their weight on driver license regardless of age group, which results in consistently lower values of BMI_D_ compared to BMI_C_. The largest difference is observed among men between the ages of 35 and 44 (BMI_D_ − BMI_C_ = − 1.38 kg/m^2^) and the smallest differences are found among those between the ages of 16 and 24 (BMI_D_ − BMI_C_ = − 0.62 kg/m^2^) and over the age of 65 (BMI_D_ − BMI_C_ = − 0.81 kg/m^2^). The magnitude of difference between self-reported and clinically measured height, weight and BMI values is smaller for men than for women.

Women at the lower end of the BMI range, as indicated by clinically measured height and weight values, have the smallest average difference between height_D_ and height_C_ (height_D_ − height_C_ = 0.08 cm for women classified as underweight), and those at the higher end of the BMI range have the highest difference (height_D_ − height_C_ = 0.42 cm for women classified as class II/III obese). When it comes to weight, the smallest average difference is observed among women in the normal weight range (weight_D_ − weight_C_ = − 1.57 kg), and the difference increases with increasing BMI. Consequently, the discrepancy between BMI_D_ and BMI_C_ is also lowest for women whose BMI falls within the normal weight category (BMI_D_ – BMI_C_ = − 0.60 kg/m^2^) and highest among those at the higher end of the BMI range (BMI_D_ – BMI_C_ = − 2.95 kg/m^2^ for women classified as type II/III obese). Women at the lower end of the BMI range – those classified as underweight – are an exception, as they tend to overestimate their weight and their BMI_D_ value is, on average, higher than their BMI_C_ value. The distribution of difference between self-reported and clinical values is similar for men in that the lowest differences are found among those who fall within the normal weight range (height_D_ – height_C_ = 0.42 cm, weight_D_ – weight_C_ = 0.87 kg, BMI_D_ – BMI_C_ = 0.16 kg/m^2^) and the differences increase with increasing BMI. Again, similarly to women, men at the lower end of the BMI range overreport their weight, which results in inflated value of BMI_D_. The magnitude of difference between self-reported and clinical values for these men is comparable to the difference observed for those classified as class I obese based on their BMI_C_.

Cross-classification of categorical BMI_D_ and BMI_C_, along with corresponding sensitivity and specificity statistics for each BMI_D_ category, and positive and negative predictive values are presented in Table 2. Among women, 94.4% of those categorized as class II/III obese based on their BMI_D_ also fall within this category based on their BMI_C_ (sensitivity = 0.542, specificity = 0.991). This indicates that there is a 94.4% probability that a woman classified as class II/III obese based on her BMI_D_ is also considered class II/III obese based on her BMI_C_. Based on the negative predictive value calculated for this group, 88.5% of women *not* assigned to the class II/III obesity category based on their BMI_D_ also do not fall within this category based on their BMI_C_. While we can assign a BMI category most accurately to class II/III women, the classification is least accurate for women classified as underweight based on their BMI_D_. Only 45.9% of women with BMI_D_ in the underweight range also have BMI_C_ in the underweight range (sensitivity = 0.555, specificity = 0.986). At the same time, negative predictive value is the highest for this category: 99.0% of women not considered underweight based on their BMI_D_ are also not underweight according to their BMI_C_. For the women classified as normal weight, overweight and class I obese based on their BMI_D_, we can correctly classify 66.6% (sensitivity = 0.909, specificity = 0.797), 51.9% (sensitivity = 0.537, specificity = 0.826) and 48.5% (sensitivity = 0.403, specificity = 0.898), respectively.

**Table 2:** Cross-classification of BMI_D_ and BMI_C_ for standard BMI categories

	*BMI*_*C*_ *(%)*				
	Underweight	Normal weight	Overweight	Class I obesity	Class II/III obesity
*Female*					
*BMI*_*D*_ *category*					
Underweight	*106 (45.9)*	117 (50.6)	7 (3.0)	1 (0.4)	0 (0.0)
Normal weight	76 (2.0)	*2491 (66.6)*	959 (25.6)	185 (4.9)	30 (0.8)
Overweight	7 (0.3)	127 (5.3)	*1239 (51.9)*	785 (32.9)	229 (9.6)
Class I obesity	2 (0.1)	6 (0.4)	94 (6.6)	*692 (48.5)*	634 (44.4)
Class II/III obesity	0 (0.0)	1 (0.1)	7 (0.6)	55 (4.9)	*1055 (94.4)*
Sensitivity	0.555	0.909	0.537	0.403	0.542
Specificity	0.986	0.797	0.826	0.898	0.991
Pos. predictive value	0.459	0.666	0.519	0.485	0.944
Neg. predictive value	0.990	0.951	0.836	0.863	0.885
*Male*					
*BMI*_*D*_ *category*					
Underweight	*25 (49.0)*	22 (43.1)	4 (7.8)	0 (0.0)	0 (0.0)
Normal weight	22 (1.1)	*1486 (73.0)*	484 (23.8)	41 (0.2)	2 (0.1)
Overweight	1 (0.0)	219 (7.2)	*2130 (70.3)*	637 (21.0)	45 (1.5)
Class I obesity	0 (0.0)	11 (0.7)	133 (8.2)	*1064 (65.4)*	420 (25.8)
Class II/III obesity	0 (0.0)	0 (0.0)	11 (1.2)	72 (7.8)	*842 (91.0)*
Sensitivity	0.521	0.855	0.771	0.587	0.643
Specificity	0.997	0.908	0.816	0.904	0.987
Pos. predictive value	0.490	0.730	0.703	0.654	0.910
Neg. predictive value	0.997	0.955	0.864	0.876	0.931

*Note. Subscript*_*C*_ *is used to denote BMI calculated using the clinical height and weight values. Subscript*_*D*_ *is used to denote BMI calculated using the height and weight values obtained from the DLD*

Similarly, among men, the best agreement is observed in the class II/III obesity category: 91.0% of men whose BMI_D_ falls within the class II/III obesity range also have BMI_C_ values within the same range (sensitivity = 0.643, specificity = 0.987). There is also a relatively high negative predictive value for this category (0.931). The classification is least accurate for men whose BMI_D_ places them in the underweight category: 49.0% of these men are also classified as underweight based on their BMI_C_ (sensitivity = 0.490, specificity = 0.997). This category also has the highest negative predictive value: 99.7% of men with BMI_D_ not falling within the underweight range are also not considered underweight based on their BMI_C_. For men in the normal weight, overweight and class I obesity categories, percentages of individuals classified correctly are 73.0% (sensitivity = 0.855, specificity = 0.908), 70.3% (sensitivity = 0.771, specificity = 0.816) and 65.4% (sensitivity = 0.587, specificity = 0.904), respectively.

Despite the discrepancies between height and weight values obtained from the DLD and clinically measured height and weight values, as well as BMI calculated using different data sources, BMI_C_ and BMI_D_ yield similar results when used as relative risk predictors in logistic regression models (Table 3). In Models 1 and 2 we used continuous variables for BMI_C_ and BMI_D_ respectively to estimate relative risk of type II diabetes. Type II diabetes diagnosis is present in in 2603 or 29% of women and 2818 or about 37% of men in our sample. The coefficients of interest in the models are very similar, with both BMI_C_ and BMI_D_ associated with a two-fold increase in relative risk of type II diabetes for a unit increase in BMI (Model 1 RR = 2.04, 95% CI 1.96–2.12; Model 2 RR = 2.09, 95% CI 2.01–2.18). When BMI is measured using four categories (underweight, normal weight, overweight, type I obesity and type II/III obesity) in Models 3 and 4, relative risk of type II diabetes is associated with progressively higher BMI categories. In Model 3 that uses BMI_C_, the relative risks for overweight, type I obesity and type II/III obesity are 1.82 (95% CI: 1.62–2.03), 3.47 (95% CI 3.09–3.90) and 6.78 (95% CI 6.02–7.63), respectively, compared to the reference category – individuals whose BMI falls within a normal weight range. Model 4, which analyzes BMI_D_ shows similar results, with relative risks of 2.15 (95% CI 1.94–2.37), 4.18 (95% CI 3.74–4.67) and 8.07 (95% CI 7.13–9.14) for individuals in overweight, type I obesity and type II/III obesity categories, respectively. In both Model 3 and Model 4, underweight individuals have lower relative risks of type II diabetes compared to the reference category. Relative risks of type II diabetes estimated using BMI_C_ and BMI_D_ for underweight individuals are 0.54 (95% CI 0.33–0.89) and 0.58 (95% CI 0.36–0.92) respectively.

**Table 3:** Estimation of relative risks of type II diabetes using continuous and categorical values of BMI_C_ and BMID

	Model 1				Model 2				Model 3				Model 4			
	Parameter Estimate	Relative Risk	95% Relative Risk Confidence Interval		Parameter Estimate	Relative Risk	95% Relative Risk Confidence Interval		Parameter Estimate	Relative Risk	95% Relative Risk Confidence Interval		Parameter Estimate	Relative Risk	95% Relative Risk Confidence Interval	
BMI_C_	0.71***	2.04	1.96	2.12												
BMI_D_					0.74***	2.09	2.01	2.18								
*Categorical BMI*_*C*_																
Underweight									−0.61*	0.54	0.33	0.89				
Overweight									0.60***	1.82	1.62	2.03				
Type I obesity									1.25***	3.47	3.09	3.90				
Type II/III obesity									1.91***	6.78	6.02	7.63				
*Categorical BMI*_*D*_																
Underweight													−0.55*	0.58	0.36	0.92
Overweight													0.76***	2.15	1.94	2.37
Type I obesity													1.43***	4.18	3.74	4.67
Type II/III obesity													2.09***	8.07	7.13	9.14

*Note. Subscript*_*C*_ *is used to denote BMI calculated using the clinical height and weight values. Subscript*_*D*_ *is used to denote BMI calculated using the height and weight values obtained from the DLD. Continuous BMI*_*C*_ *and BMI*_*D*_ *included in Models 1 and 2 were scaled. Normal weight (BMI between 18.5 and 24.9 kg/m* ^*2*^ ) is a reference category in Models 3 and 4. All models include birth year, sex, education, race and ethnicity as additional covariates (estimates not shown). Significant results are indicated as follows: * p ≤ 0.05, ** p ≤ 0.01, ***p ≤ 0.001

## Discussion

In our sample, self-reported height and weight differ from clinically measured values in a predictable manner: individuals, on average, overestimate their height and underestimate their weight, resulting in significant differences between BMI_D_ using height and weight values from the driver license and BMI_C_ using clinically measured height and weight. For women, the difference between BMI_D_ and BMI_C_ is equal to − 2.19 kg/m^2^, and for men, the difference is equal to − 1.06 kg/m^2^. These results are consistent with previous findings indicating consistent underestimation of BMI based on self-reported height and weight values [21, 22, 24, 25, 30, 31, 35].

The discrepancy between BMI_D_ and BMI_C_ is significant across age and BMI_C_ categories, although there is variation between groups. Among women, the difference between BMI_D_ and BMI_C_ values is greatest between the ages of 25 and 34, and among men it is greatest between the ages of 35 and 44. For women, the average difference between BMI_D_ and BMI_C_ decreases with age, while the relationship between age and BMI discrepancy for men is U-shaped. The smallest average BMI discrepancies are found for women aged 65 and older and, among men, for those aged 16 to 24 and aged 65 and older. Previous studies suggest that the discrepancy between self-reported and clinically measured BMI value increases with age [19, 20], which differs from our results. When it comes to distribution of discrepancy across BMI_C_ values, the largest differences between BMI_D_ and BMI_C_ are found at the extreme end of the BMI_C_ scale: BMI_D_ − BMI_C_ is equal to − 4.48 kg/m^2^ for class II/III obese women and − 1.32 kg/m^2^ for class II/III obese men. This finding is consistent with previous studies showing the greatest weight and BMI underestimation at higher values of BMI [7, 20, 22, 23, 35–37].

BMI_D_ obtained from driver license records allows for fairly accurate classification into BMI_C_ category for class II/III obese individuals. Although classification is less successful in the remaining BMI categories, the pattern of misclassification exhibits regularity: for each category, those that are misclassified tend to fall in the next highest category. For example, 66.6% of women who are considered normal weight based on their BMI_D_ are also assigned into the normal weight category based on their BMI_C_. Of those that remain, the majority are assigned into the overweight category – one category above normal weight. Similarly, we can correctly classify 51.9% of women whose BMI_D_ falls within the overweight range, and the majority of those not classified correctly are in the class I obesity category based on their BMI_C_. Because of this misclassification pattern, we can accurately classify individuals not only to the class II/III obesity category, but also to a combined obesity category, which includes class I and class II/III obese individuals (BMI ≥ 30.0 kg/m^2^). When treating class I and class II/III obesity as separate categories, we are able to correctly classify 48.5% of women and 65.4% of men whose BMI_D_ falls within the class I obesity range. However, for both men and women, the majority of those misclassified are in the class II/III obesity category. Consequently, when combining the two categories we can achieve positive predictive values of 0.957 for women and 0.939 for men. We conclude that although BMI_D_ does not allow for very accurate classification of individuals in the underweight, normal weight and overweight categories, it can be particularly useful for BMI categorization at the high end of the BMI scale.

To assess predictive utility of BMI_D_ and BMI_C_, we estimated relative risks of type II diabetes using continuous and categorical versions of BMI_D_ and BMI_C_. In models with continuous predictors, relative risk estimates associated with BMI_D_ and BMI_C_ are remarkably similar: an equal increase in BMI_D_ and BMI_C_ is associated with a two-fold increase in relative risk of type II diabetes. When treated as categorical predictors, BMI_D_ and BMI_C_ also behave similarly: those in the underweight category experience reduction of relative risks of having a condition relative to those classified as normal weight, and risks are progressively greater in the overweight, class I obese and class II/III obese individuals. For these three categories, relative risks estimated using BMI_D_ are somewhat higher compared to those estimated using BMI_C_, but 95% confidence intervals overlap. Larger relative risk estimates for the model with a categorical BMI_D_ predictor can be partially explained by the pattern of BMI misclassification observed in the data. Our models indicate that BMI_D_ obtained from driver license records is comparable to clinically measured BMI_C_ when used as a predictor of type II diabetes. Furthermore, relative risks estimates calculated using BMI_C_ are more conservative compared to those calculated using BMI_D_. Comparable analyses of other health outcomes that are associated with BMI and with data from different populations can help further validate the value of using driver license data for assessing health risks.

It is important to acknowledge that although we assume that clinically recorded height and weight values more accurately reflect individuals’ true height and weight, clinical records are susceptible to measurement error as well. It is not possible to determine whether the patients were asked to remove shoes and clothing when their height and weight were recorded. In addition, one must be cautious when generalizing results of the present study to other populations, in particular, populations with greater degree of racial and ethnic diversity. Individuals in our sample are predominantly white, and height and weight misreporting vary by ethnicity [7, 20, 23, 36, 37]. Finally, by relying on a major medical provider as a source of clinical height and weight measurements, we are likely systematically omitting a portion of the population with limited access to health services, i.e. un- and under-insured and lower income individuals. Socioeconomic status may influence one’s perception of own body, which, in turn, can affect the degree of weight misreporting [37].

## Conclusions

We demonstrate that self-reported height and weight data obtained from the driver license records differ systematically from clinically measured height and weight. The differences result in BMI calculated using the driver license data being lower than clinically measured BMI. BMI based on driver license records allows for accurate classification of individuals for those categorized as obese, and performs similarly to clinically measured BMI as a predictor of relative risk of type II diabetes mellitus. We conclude that driver license height and weight data can be a useful asset for monitoring population health. States that do not currently collect height and weight information during the driver license application process may consider establishing a procedure for doing so, as it would allow for more efficient monitoring of population health.

## Abbreviations

BMI: Body Mass Index
BRFSS: Behavioral Risk Factor Surveillance System
DLD: Driver License Division
UPDB: Utah Population Database
UUHSC: University of Utah Health Science Center
